# α7nAchR/NMDAR coupling affects NMDAR function and object recognition

**DOI:** 10.1186/1756-6606-6-58

**Published:** 2013-12-20

**Authors:** Shupeng Li, Qiang Nai, Tatiana V Lipina, John C Roder, Fang Liu

**Affiliations:** 1 Department of Neuroscience, Centre for Addiction and Mental Health, Clarke Division, 250 College Street, Toronto, Ontario M5T 1R8, Canada; 2 Samuel Lunenfeld Research Institute at Mount Sinai Hospital, Toronto, Ontario M5T 1R8, Canada; 3 Departments of Psychiatry, University of Toronto, Toronto, Ontario M5T 1R8, Canada

**Keywords:** α7nAchR, NMDAR, Learning, Memory, LTP

## Abstract

The α7 nicotinic acetylcholine receptor (nAchR) and NMDA glutamate receptor (NMDAR) are both ligand-gated ion channels permeable to Ca^2+^ and Na^+^. Previous studies have demonstrated functional modulation of NMDARs by nAchRs, although the molecular mechanism remains largely unknown. We have previously reported that α7nAchR forms a protein complex with the NMDAR through a protein-protein interaction. We also developed an interfering peptide that is able to disrupt the α7nAchR-NMDAR complex and blocks cue-induced reinstatement of nicotine-seeking in rat models of relapse. In the present study, we investigated whether the α7nAchR-NMDAR interaction is responsible for the functional modulation of NMDAR by α7nAchR using both electrophysiological and behavioral tests. We have found that activation of α7nAchR upregulates NMDAR-mediated whole cell currents and LTP of mEPSC in cultured hippocampal neurons, which can be abolished by the interfering peptide that disrupts the α7nAchR-NMDAR interaction. Moreover, administration of the interfering peptide in mice impairs novel object recognition but not Morris water maze performance. Our results suggest that α7nAchR/NMDAR coupling may selectively affect some aspects of learning and memory.

## Background

Glutamate is the principal excitatory neurotransmitter in brain and N-methyl-D-aspartate (NMDA) receptors, one of the major glutamate receptors, are important in the activity-dependent synaptic plasticity and excitotoxicity that underlies learning, memory, neural development and some neurological disorders [1-3]. Both NMDAR and the α7 nicotinic acetylcholine receptor (nAchR) are ligand-gated ion channel receptors with high Ca^2+^ permeability. NMDARs contain intrinsic ion channels comprised of NR1 subunits, an essential subunit of NMDAR that exists as a number of splice variants, and NR2 subunits, which are encoded by four different gene products, termed NR2A-D [4,5].

Nicotine interacts with nicotinic receptors (nAchRs) in the brain to initiate neuroadaptive changes at both cellular and circuit levels. The nAchRs are composed of five distinct membrane-spanning subunits (α and β subunits) that combine to form a functional receptor. There are nine isoforms of the neuronal α subunit (α2–β10), and three isoforms of the neuronal β subunit (β2–β4) [6]. Various sub-types of nAchRs differ in their subunit composition and sensitivity to nicotine and are expressed in addiction-relevant brain regions including prefrontal cortex, nucleus accumbens, dorsal striatum, and hippocampus [7]. Unlike NMDARs, nAchRs can exist as both hetero-metric and homo-metric- assemblies of these subunits. α7 nAchRs are highly expressed in hippocampus [7].

The activation of nAchRs can modulate glutamatergic neurotransmission in several ways. Previous studies have reported that nicotine facilitates the induction of LTP in the hippocampal CA1 region [8] by the activation of α7 nAchRs on pyramidal cells [9,10]. This induction of LTP can be blocked by AP5, an NMDAR antagonist [11]. Furthermore, *in vivo* nicotine exposure was reported to induce the enhancement of NMDAR currents in the hippocampus [12]. This nicotine effect is maintained during continued nicotine exposure and is accompanied by increased tyrosine phosphorylation of NR2B [13]. In contrast to the presynaptic nAchRs, somatic or postsynaptic nAchRs can initiate a Ca^2+^ signal that can act via calmodulin to reduce the responsiveness of NMDARs, as manifested by evoked excitatory postsynaptic currents (eEPSCs) [14]. Furthermore, NMDAR antagonists have been found to interfere with tolerance, sensitization, physical dependence and conditioning to self-administrated nicotine, as well as other drugs of abuse [15].

We have previously shown that the α7nAchR interacts with NMDARs and their coupling mediates cue-induced reinstatement of nicotine in rat [16]. In the present study, we plan to investigate the role of α7nAchR-NMDAR coupling in modulating NMDAR functions. Since both α7nAchR and NMDAR have been implicated in learning and memory, we will also investigate the behavioral effects of α7nAchR-NMDAR coupling in some cognitive tests.

## Results and discussion

### Activation of α7nAchR increases NMDAR mediated whole-cell currents

**Figure 1:**
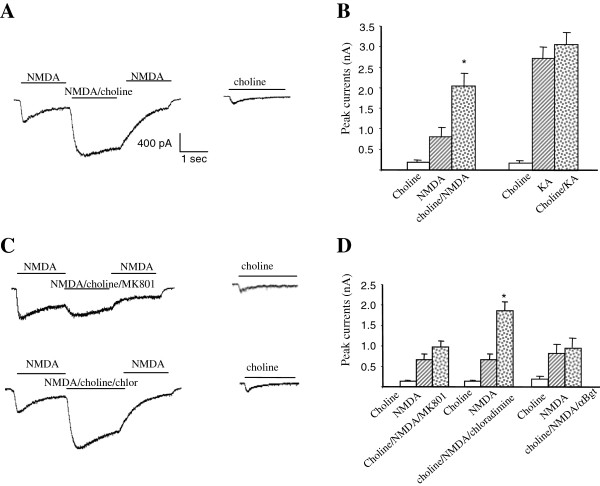
**Choline induced synergistic effect on NMDAR currents through the α 7-nAchR/NMDAR direct protein-protein interaction. (A)** Co-application of 1 mM choline with 50 µM NMDA/10 µM glycine produced a synergistic effect that display a significantly larger current compared to the current induced by NMDA/Glycine alone (n = 43 of 47 cells, P < 0.01). **(B)** The choline induced synergistic effect is specific to NMDAR-mediated currents since no such an effect was detected on currents induced by 100 µM kainic acid. **(C, D)** The choline-induced synergistic effect is significantly inhibited by simultaneous application NMDAR channel blocker MK-801 (10 µM) (n = 8, p < 0.05), but not the nAchR channel blocker chlorisondamine (20 µM). Furthermore, pretreatment of the neurons with the α7nAchR specific antagonist α-Bungarotoxin for 40 minutes inhibited the choline-induced synergistic effect.

Previously, we showed that activation of α7nAchR by choline facilitates α7nAchR-NR2A complex formation [16]. To assess the functional impact of the α7nAchR-NMDAR interaction following α7nAchR activation, we examined the effects of α7nAchR activation on NMDAR-mediated whole-cell currents in rat hippocampal primary cultures. As shown in Figure 1A, co-application of 1 mM choline with 50 μM NMDA/10 μM glycine produced a significantly larger current than the current induced by NMDA/Glycine alone (choline/NMDA/glycine: 2036.3 ± 317.2 pA; NMDA/glycine: 812.9 ± 215.5 pA, n = 43, p < 0.05). The synergistic effect of choline/NMDA co-application is specific to NMDAR since co-application of choline with 100 μM KA did not enhance whole-cell currents compared to KA treatment alone (Figure 1B).

It is difficult to differentiate whether the observed enhancement of whole-cell current induced by co-application of choline with NMDA is mediated by nicotinic receptors or NMDARs since both receptors are cation ion channel that are permeable to calcium and sodium. However, the observed enhancement of whole cell current induced by co-application of choline with NMDA can be blocked by simultaneous application of the NMDAR channel blocker MK-801 (10 μM), but not with the nicotinic receptor open channel blocker chlorisondamine (20 μM) (Figure 1C, D). This suggests that the observed enhancement of whole cell currents is due to ion influx through NMDAR, but not nicotinic receptors. Furthermore, α7-nAchR specific antagonists α-bungarotoxin abolish the synergistic effect of choline/NMDA co-application (Figure 1D), indicating that the activation of α7-nAchR is required for this process.

### Activation of α7nAchR facilitates NMDAR dependent LTP of mEPSCs

**Figure 2:**
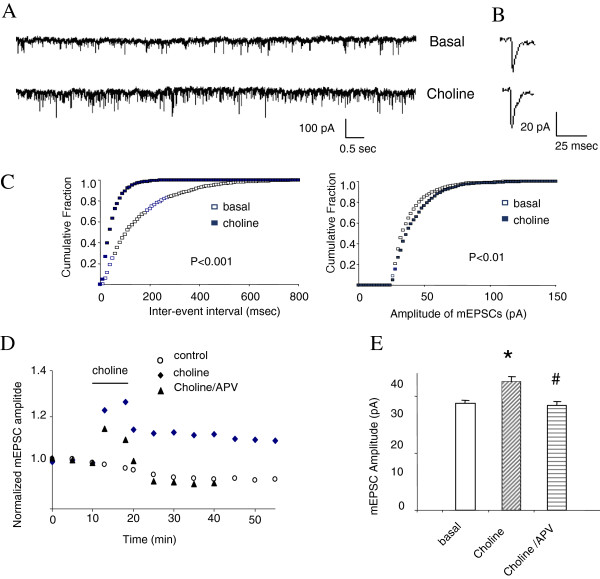
**Choline induced upregulation of NMDAR-dependent LTP of mEPSCs in cultured hippocampal neurons. (A)** Examples of continuous recordings from individual neurons 5 minutes before (Basal) and 30 minutes after 8-minute stimulation of neurons with 1 mM choline. **(B)** Single events taken from the basal and choline traces, respectively showing that the amplitude of mEPSCs was increased by choline application. **(C)** Cumulative fraction plots for mEPSCs inter-event intervals and amplitudes obtained 5 minutes before (Basal) and 30 minutes after choline (8 min, 1 mM). **(D)** mEPSC amplitudes are normalized to the values from the initial 10 min and plotted over time. Treatment of neurons with choline (8 min, 1 mM) significantly increased the amplitude of the mEPSCs over the time course of recordings; an effect can be abolished by NMDAR antagonist, AP5 (100 μM). **(E)** Amplitude histogram summarizes data from groups of individual neurons treated with glycine (200 μM; 3 min) in the absence or presence of choline (1 mM) or choline/AP5 (100 μM). Responses obtained 30 min after glycine treatment (26.5+/- 2.3 pA), 30 min after choline treatment (31.4 +/-2.7 pA, n = 6, *p < 0.01) and 30 minutes after coapplication of choline/APV (25.9+/-2.0pA n = 3, **p < 0.05, paired t-test).

To determine whether the α7nAchR is able to regulate synaptic strength, we examined the miniature excitatory postsynaptic currents (mEPSCs) during LTP upon activation of α7nAchR. Previous studies have demonstrated that activation of nicotinic acetylcholine receptors facilitates induction of long-term potentiation, although the molecular mechanism underlying this process remains unknown. Thus, we initiated our investigation by confirming the effect of nicotine on mEPSC during LTP, using the glycine-induced LTP model in rat hippocampal primary neuron cultures. The glycine-induced LTP model is similar to the electrically evoked EPSCs in CA1 neurons in hippocampal slices [17-19]. Consistent with previous studies in brain slices, choline application (1 mM, 8 minutes) significantly enhanced the frequency of mEPSC during LTP produced by glycine application (200 μM; 3 min) (Figure 2A, C). There is only a small but significant increase in current amplitude mEPSC of LTP (Figure 2B-E), which may reflect the nature of LTP in primary cultures and the recording paradigm [20,21]. We also concluded that the choline-induced upregulation of mEPSC of LTP is NMDAR dependent since D-APV (100 μM) co-applied with choline blocked the effect of choline on both the frequency (data not shown) and the amplitude (Figure 2E) mEPSC of LTP.

### α7nAchR-NMDA coupling is responsible for modulation of NMDAR function by the activation of α7nAchR

**Figure 3:**
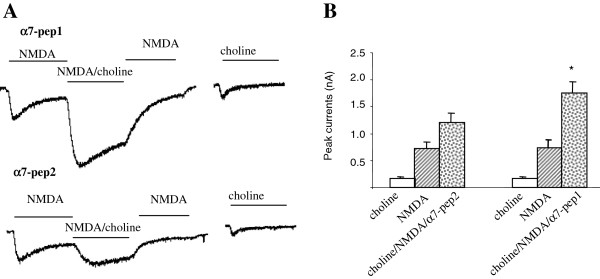
**Application of α7pep2 peptide blocked choline induced upregulation of NMDA current in hippocampal primary culture. (A)** The choline-induced synergistic effect is significantly inhibited by intracellular application of interfering peptides α7pep2, but not α7pep1 (10 µM) (choline/NMDA: 1202.7 ± 182.1 pA; NMDA: 910.5 ± 130.8, n = 6, p > 0.05). Cells were hold at -70 mV, 20 mM bicuculline, 1 mM strychnine, 0.5 μM TTX, 1 mM glycine were included in the extracellular solution. **(B)** Amplitude histogram summarizes data from groups of individual neurons treated with glycine (200 μM; 3 min) in the absence or presence of choline (1 mM) with the intracellular application of α7pep1, α7pep2 peptide respectively. Responses obtained 30 min after glycine treatment (basal) and 30 min after choline treatment (choline). α7pep1 peptide did not block the enhancing effect of choline on the mEPSC amplitude (basal: 25.2 +/-2.1 pA; choline: 28.4+/-2.4 pA, n = 4, *p < 0.01, paired t-test) while choline failed to upregulate mEPSC amplitude with the presence of α7pep2 (basal: 24.2+/-2.0; choline: 25.1 +/-2.3 pA, n = 6, p > 0.05, paired t-test).

Next, we determined whether the direct coupling of α7nAchR-NMDA plays a role in the functional interaction between α7nAchR and NMDAR. Our previous reports showed α7nAchR/NMDAR coupling was mediated by a 10 amino acid fragment (L336-M345) within the second intracellular loop of α7nAchR. Administration of this peptide could disrupt α7nAchR/NMDAR coupling as shown in the co-immunoprecipitation experiment. Furthermore, this peptide blocked cue-induced nicotine reinstatement in an animal model of relapse [16]. As shown in Figure 3A, B, intracellular application of α7pep2[L_336_-M_345_] peptide (10 μM), which has been shown to be able to disrupt α7nAchR-NMDA coupling, blocked the choline-induced enhancement of NMDA-mediated whole cell currents, while the control peptide, α7pep1[R_316_-G_325_], has no such effect. These data suggest that the α7nAchR-NR2A interaction is required for the functional modulation of NMDAR by the activation of α7nAchR.

**Figure 4:**
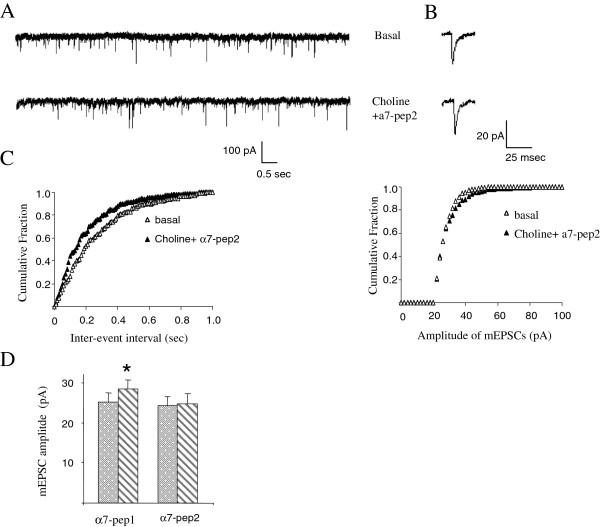
**Application of α7pep2 peptide blocked choline induced upregulation of mEPSC of LTP in hippocampal primary culture. (A)** Examples of continuous recordings from individual neurons 40 minute after intracellular application of α7pep2 peptide (10 µM) with/without the presence of choline (1 mM, 8 min). **(B)** Single events taken from the basal and choline traces after intracellular application of α7pep2 peptide, showing that choline application failed to increase the amplitude of mEPSCs. **(C)** Cumulative fraction plots for mEPSCs inter-event intervals and amplitudes obtained 5 minutes before (Basal) and 30 minutes after choline (8 min, 1 mM) with the presence of α7pep2 peptide intracellularly. **(D)** Amplitude histogram summarizes data from groups of individual neurons treated with glycine (200 μM; 3 min) in the absence or presence of choline (1 mM) with the intracellular application of α7pep1, α7pep2 peptide respectively. Responses obtained 30 min after glycine treatment (basal) and 30 min after choline treatment (choline). α7pep1 peptide did not block the enhancing effect of choline on the mEPSC amplitude (basal: 25.2 +/-2.1 pA; choline: 28.4+/-2.4 pA, n = 4, *p < 0.01, paired t-test) while choline failed to upregulate mEPSC amplitude with the presence of α 7pep2 (basal: 24.2+/-2.0; choline: 25.1 +/-2.3 pA, n = 6, p > 0.05, paired t-test).

Furthermore, we tested the effect of the interfering peptide α7pep2[L_336_-M_345_] in choline-mediated NMDAR-dependent mEPSC changes during LTP. As shown in Figure 4A-D, intracellular application of α7pep2[L_336_-M_345_] peptide blocked choline-induced upregulation of mEPSC frequency and amplitude during LTP, indicating that the α7nAchR-NR2A interaction is essential for choline-induced modulation of NMDAR-dependent mEPSCs during LTP.

### Disruption of the α7nAchR-NR2A interaction selectively impaired Novel Object Recognition

**Figure 5:**
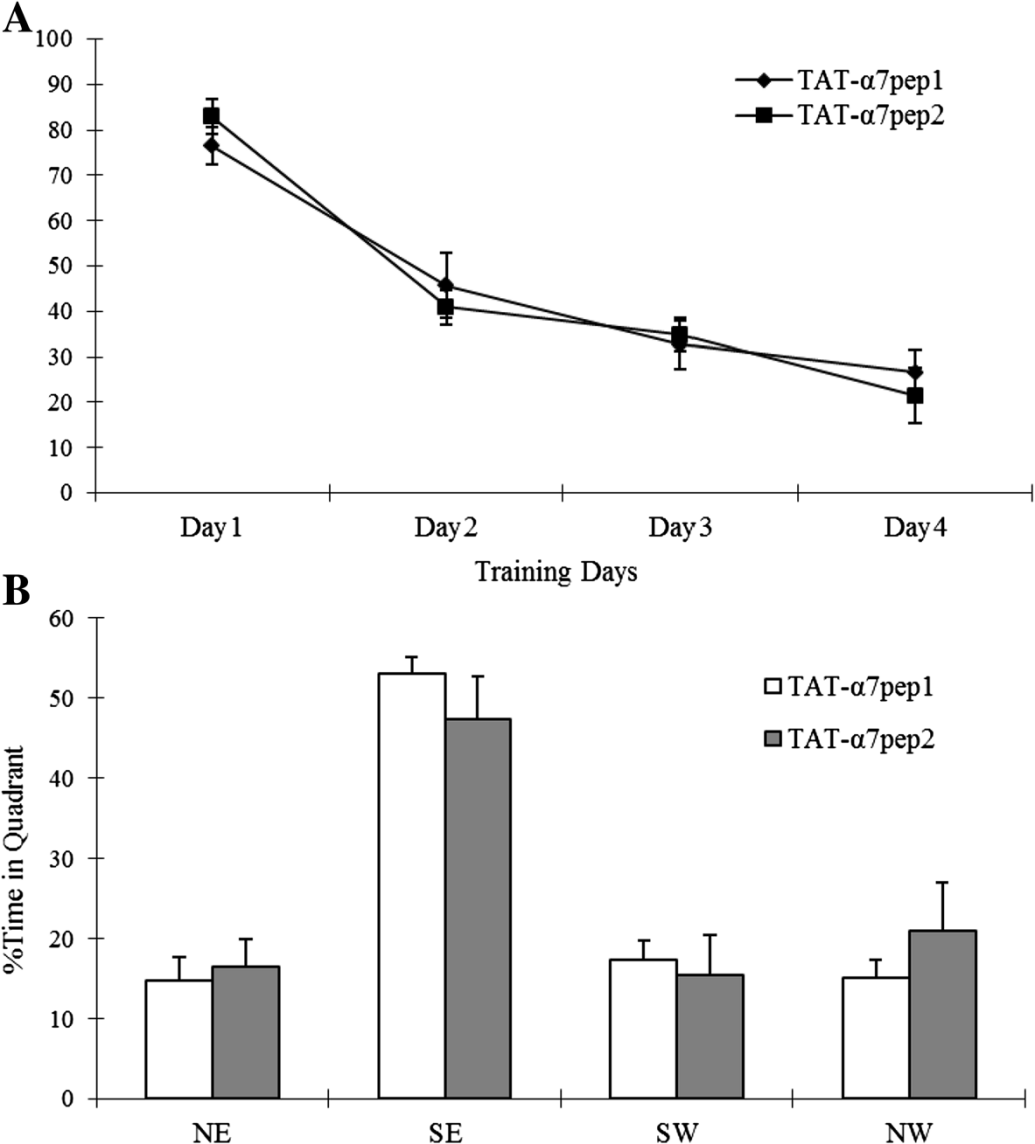
**TAT-α7pep2 peptide treatment has no effects on spatial learning and memory.** Latency to find the platform of mice was not affected by peptide treatment in the Morris water maze task. **(A)** In the acquisition phase, escape latency to find a hidden platform located in the southeast (SE) quadrant was unaffected by treatment. **(B)** Histogram of percent time spent in each quadrant at probe test.

Both α7nAchR and NMDARs have been implicated in learning and memory processes. Thus, we sought to investigate whether the α7nAchR-NR2A interaction might affect learning and memory. We first tested the α7pep2[L_336_-M_345_] peptide for possible effects on the Morris water maze. Mice were injected intraperitoneally with TAT-α7pep2[L_336_-M_345_] (3 ng/g) or TAT-α7pep1[R_316_-G_325_] 30 min prior to training and probe trials. As shown in Figure 5, there is no difference between α7pep2[L_336_-M_345_] peptide treated mice and TAT-α7pep1[R_316_-G_325_] treated mice in latency to find the platform. There is also no difference between the two groups in the acquisition phase, nor in the probe trial, indicating that the disruption of the α7nAchR-NMDAR interaction has no effect on the spatial learning and memory required for this task.

**Figure 6:**
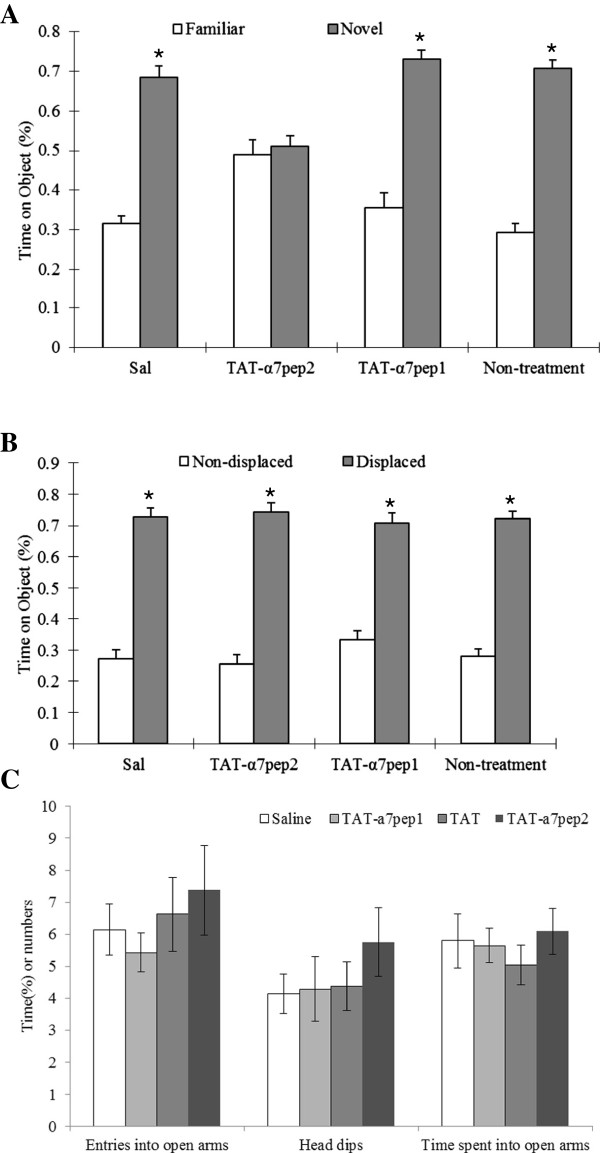
**TAT-α7pep2 Peptide treatment affected nonspatial learning and memory.** TAT-α7pep2 Peptide treatment impaies novel object recognition **(A)** but not displaced object recognition **(B)**. Times of exploration of the DO and NDO were recorded and expressed as a percentage of the total time of objects investigated. In the novel object recognition session, one of the familiar NDOs was replaced with a new object (NO) at the same location and the two familiar DOs were removed. Data were analyzed with ANOVA with treatment as a between-subjects factor, and object rearrangement or object replacement as a repeated measures factor. The Tukey test was used for post hoc comparisons when ANOVA yielded statistically significant main effects or interactions. **(C)** In the elevated plus maze, no significant changes in the percent of time spent in open arms, entries into the open arms and head dips were observed in different treatment groups.

To further evaluate the effect of our interfering peptide on cognition, we used two other behavioral tests: the displaced object recognition task and the novel object recognition task. As shown in Figure 6 A, TAT-α7pep2[L_336_-M_345_] peptide treatment, but not TAT-α7pep1[R_316_-G_325_] treatment, induces impairment in novel object recognition in mice. In contrast, there is no difference between the two groups in the displaced object recognition task (Figure 6B). To investigate whether the TAT-α7pep2[L_336_-M_345_] peptide might affect anxiety-related behaviour, we tested the effect of TAT-α7pep2[L_336_-M_345_] in the elevated plus maze. As shown in Figure 6C, there is no difference between the two groups in the number of entries into the open arms, the time spent on the open arms and the head dips. There is also no difference between the two groups in the total distance travelled, margin and central distance travelled, and time spent in the marginal and central zones (Additional file 1: Figure S1A-E). Taken together, our findings suggest that the α7nAchR-NMDAR interaction may selectively impair novel object recognition.

In the present study, we provide evidence that the α7nAch-NMDAR complex modulates NMDAR-mediated whole cell currents and LTP. Furthermore, disruption of this complex via an interfering protein peptide TAT-α7pep2 [L_336_-M_345_] had no effect on Morris water maze and displaced object recognition in mice, but specifically impaired novel object recognition. Our study provide the first demonstration that α7nAchR regulates NMDA-mediated whole cell currents and LTP through a protein-protein interaction. More interestingly, our data suggest that the α7nAchR-NR2A interaction may specifically play a role in non-spatial learning and memory.

Regulation of ligand-gated ion channel function was traditionally thought to be mainly regulated by receptor phosphorylation and trafficking [22,23]. Receptor phosphorylation involves intracellular second-messengers, including various phosphatase/kinases, while receptor trafficking can be induced by either receptor phosphorylation or direct coupling with intracellular proteins that lead to changes in receptor conformation or receptor plasma membrane expression. Thus, receptor phosphorylation, conformational changes and plasma membrane expression constitute the major means to modulate ligand-gated ion channel function [24].

We did not directly investigate the mechanism by which the α7nAchR-NR2A interaction can lead to enhanced NMDA currents. However, we speculate that there are several possible mechanisms: enhanced phosphorylation, conformational changes or altered cell surface expression of NMDAR. Currently, there is no evidence supporting that either α7nAchR or NMDAR are able to directly activate second messenger systems, however, both receptors are calcium permeable [25]. It is possible that the calcium influx induced by the activation of α7nAchR may lead to the activation of intracellular signaling pathways that lead to changes in NMDAR phosphorylation, with potential downstream functional changes. The α7nAchR-NMDAR complex may also induce conformational changes in the NMDAR or enhance NMDAR cell surface expression that could alter current flow.

We have found that the administration of the interfering peptide in mice impairs novel object recognition, but not Morris water maze performance and displaced object recognition, suggesting that the α7nAch-NR2A interaction may specifically play a role in non-spatial learning and memory. However, the fact that our interfering peptide blocks choline-induced upregulation of mEPSC during LTP, which contributes to spatial learning performance, seems contradictory. LTP has been recognized as a cellular model for learning and memory. Although LTP is generally thought to be associated with spatial learning and memory, there are examples of inconsistencies between LTP and Morris water maze performance, a common way of testing spatial learning and memory. For example, Dr. Morris’ lab reported in 1995 [26] that AP5 (an NMDA receptor antagonist) impaired both LTP and water maze performance. However, the AP5-induced learning deficit in the water maze can be prevented if rats are pre-trained in a different water maze before administration of AP5, implying that NMDA receptor-mediated LTP may not be required for all components of spatial learning. In addition, the same issue of Nature includes another paper by Saucier and Cain that shows NMDA receptor-mediated dentate LTP is not required for normal spatial learning in the water maze [27]. Thus, our result showing that disruption of α7nAch-NR2A interaction impairs LTP, but not Morris water maze performance, seems contradictory, but it is not unique.

We have previously found that disruption of α7nAchR-NMDAR complex using TAT-α7pep2[L_336_-M_345_] blocks cue induced reinstatement of nicotine self-administration in rats [16]. In the current study, we have further explored behavioral changes in mice given TAT-α7pep2[L_336_-M_345_]. We found that TAT-α7pep2[L_336_-M_345_] had no effect on spatial learning and memory in the Morris water maze and displaced object recognition task in mice, but did affect novel object recognition. This is consistent with previous findings that systemic administration of selective α7nAchR agonists reverse working memory impairments caused by NMDAR blockade in several behavioral tasks including the 16-arm radial maze, Y-maze, Morris water maze and linear maze, and novel object recognition test [28-31]. There are also other examples of functional interaction between the α7nAchR and NMDAR. Cholinergic innervation of the hippocampus modulates activity-dependent synaptic plasticity, such as long-term potentiation (LTP) and other processes that contribute to learning and memory [32]. Nicotine was found to enhance LTP of EPSPs in the dentate gyrus and to convert weak stimuli-evoked short-term potentiation into LTP in the CA1. The selective α7 nAchR agonists choline and 2,4-dimethoxybenzlidine anabaseine have also been found to mimic the facilitative action of nicotine in potentiating LTP [33-35], although the mechanisms underlying the effects of α7nAchR on NMDAR-mediated function remain unclear.

## Conclusions

Our results confirm a physical interaction between α7nAchR and the NR2A of NMDAR that affects both NMDAR-mediated function and novel object recognition. These findings increase our understanding of these two receptor systems and suggest future experiments to further investigate the mechanisms underlying the functional effects of the interaction.

## Methods

### Primary cultures of dissociated cells

Hippocampi were collected from fetal (E18) Wistar rats. Fetuses are removed from pregnant rats anesthetized by inhalation of isoflurane and killed by cervical dislocation. The dissection and dissociation were performed in ice-cold Hank’s balanced salt solution (HBSS, without Ca^++^ and Mg^++^ Gibco) supplemented with 10 mM HEPES (pH 7.4) and 1 mM sodium pyruvate. Neurons were mechanically dispersed by trituration using glass Pasteur pipettes with reduced tips and then added to plating solution composed of 89.5% Neural Basal (NB), 10% horse serum, and 0.5% Penicillin/streptomycin (P/S) [36]. The cells were plated on German origin glass coverslips coated with 0.1 mg/ml poly-d-lysine in Borate Buffer. The cell density was about 50,000-80,000/ml. After 5/6 hours of plating, half of the plating solution was replaced by feeding solution containing 98% NB, 2% B-27 supplement, 0.5 mM L-glutamine and 0.5% P/S (all from Gibco). The cultures were maintained by feeding twice weekly by replacing half of the solution with fresh feeding solution. After 6 days of plating, 5 μM Ara-C was added to stop the growth of glial cells.

### Electrophysiology

Miniature excitatory postsynaptic currents (mEPSCs) were recorded from cultured hippocampal neurons 2 to 4 weeks days after plating under a whole-cell patch clamp configuration [36]. Electrodes (3–5 MΩ) were pulled from high lead pipettes (Corning 8161, Warner Instruments). Cells were voltage clamped at -70 mV. Access resistance is below 10 MΩ; recordings with access resistance varying more than 10% were rejected from analysis. The extracellular solution contained (in mM) NaCl 140, CaCl_2_ 1.3, KCl 5.0, HEPES 25, glucose 33, TTX 0.0005, strychnine 0.001, and bicuculline methiodide 0.01, at pH 7.4 and osmolarity 325–335 mosmol^-1^. Each of the tested cells was continuously perfused with the extracellular solution from a single barrel of a computer-controlled multi-barreled fast-step perfusion system (Warner Instruments Inc.). The receptor agonists were applied from different barrel(s).

The response to nicotinic agonists by different hippocampal cultures was variable. Overall, about 30% (53 of 170 cells) of the cells displayed positive nicotinic responses (more than ten times the basal RMS noise level). Only responsive cells were used for further whole-cell or synaptic activity analysis. The intracellular solution consisted of (in mM): CsCl_2_ 140, EGTA 2.5, MgCl_2_ 2, HEPES 10, TEA 2, and K_2_ATP 4, at pH 7.3; and osmolarity 300 to 310 mosmol^-1^[19]. In some experiments 10 μM of peptides α7pep1 and α7pep2 were included in the intracellular solution and dialyzed for 30 minutes before recording. Recordings were made at room temperature (21-23°C). Series resistance was not compensated. Synaptic activity was recorded using an Axopatch 200B (Axon Instruments, Inc.); signals were filtered at 2 kHz, digitized at 10 kHz, and stored in a lab computer. Data were analyzed using Mini Analysis Software (Synaptosoft, Inc.). mEPSC frequency and amplitude for each time point were obtained from a two minute recording. The trigger level for event detection was three times higher than that of baseline noise. Visual inspection was performed to eliminate false events. Data were expressed as mean ±SEM, t-test were used to test the statistical significance of differences between groups.

### Behavioural testing

All animal procedures were conducted in accordance with the requirements of the Province of Ontario Animals for Research Act, 1971 and the Canadian Council on Animal Care (CCAC 1984, 1995). To examine the effects of α7pep2[L_336_-M_345_] in learning and memory, C57BL/6 J mice were used for the water maze and object recognition tasks. In the water maze task, a single daily intraperitoneal injection of α7pep2[L_336_-M_345_] (3 ng/g) or vehicle was administered 30 min prior to training and on the probe trial day. For the object recognition task, a single intraperitoneal injection of α7pep2 or vehicle was administered 30 min prior to object recognition testing.

Locomotor activity was monitored in a directly illuminated (600 lux) clear Perspex chamber (42 × 42 × 30 cm; Accuscan Instruments Inc., Columbus, OH, USA) by interruptions of 16 horizontal and 16 vertical sensors (infrared beams) spaced 2.5 cm apart. Data was recorded every 5 min of the testing period. Data were analyzed with two way analyses of variance (anovas) with treatment as main factor and repeated measures (time intervals).

For the Morris water maze task, 12–16-wk-old C57BL/6 J mice were used. The water maze consisted of a 185 cm diameter cylindrical tank containing a 15 cm circular platform and water (26 ± 1°C) rendered opaque by the addition of white non-toxic paint. The training regime consisted of acquisition training to a hidden platform in the southeast (SE) quadrant for 3 d (day 1–3; six trials per day; maximum duration, 90 s; ITI, 40 min). Probe trials (90 s duration) were administered 18 h after the last acquisition. All behavioural events were video recorded and analyzed using Observer 5.0 software (Noldus Information Technology). Behavioural data for escape latency were analyzed using a two-way ANOVA with training days as repeated measurement. For the probe trials, statistical comparisons between groups for the time over quadrants were done using one-way ANOVA with the critical α level set to 0.05 for all statistical analyses.

Object recognition tests were performed as described [37] using a modified open field set-up. The open field apparatus consisted of a square box (41 × 41 × 33 cm) made of clear Perspex (Ugo Basile) that was connected to horizontal and vertical infrared sensors. During the habituation session, four different plastic objects were presented in the open field: a cube (5 × 5 × 5 cm), hollow cylinder (6 cm height and 4 cm diameter), solid cylinder (3 cm height × 6 cm diameter), and prism (3.5 × 4.5 × 6 cm). Exploration of the four different plastic objects in the open field were measured every 5 min for 15 min under dim lighting (habituation).

In the displace object recognition session, the four objects, initially placed in a square arrangement, were reconfigured into a polygon-shaped pattern by moving two objects (the displaced objects or DO). The remaining two objects were left at the same location (nondisplaced objects [NDOs]). Times of exploration of the DO and NDO were recorded for 5 min and expressed as a percentage of the total time spent investigating objects. In the novel object recognition session, one of the familiar NDOs was replaced with a new object (NO) at the same location and the two familiar DOs were removed. The time examining a NO or a familiar object (FO) was recorded for 5 min and was expressed as a percentage of the total time spent investigating objects. Data were analyzed using ANOVA with drug treatment as a between-subjects factor, and object rearrangement or object replacement as a repeated measures factor. The Tukey test was used for post hoc comparisons when the ANOVA yielded statistically significant main effects or interactions.

### Elevated plus maze (EPM)

Experiments were conducted in a dimly lit room with a light intensity on the central platform of 210 lux [38]. During a 5-min observation period, the number of entries (defined as four paws into a maze arm) and the amount of time spent in the open arms, closed arms and the central platform were scored by the observer. The total number of entries for each subject was collected. These data are presented as percentage time spent in closed or open arm/total duration of experiment × 100. Data were analyzed using ANOVA with drug treatment as a between-subjects factor.

## Abbreviations

EPM: Elevated plus maze; DO: Displaced objects; NDOs: Nondisplaced objects; NO: New object; FO: Familiar object; mEPSCs: Miniature excitatory postsynaptic currents; LTP: Long-term potentiation; eEPSCs: Evoked excitatory postsynaptic currents; nAchR: nicotinic acetylcholine receptor; NMDAR: N-methyl-D-aspartate receptor.

## Competing interests

The authors declare that they have no competing interests.

## Authors’ contributions

SL and TL conducted the behavior tests and data analysis; QN conducted the electrophysiology experiments and analyzed all the electrophysiology data. JR advised the design of the behavior tests. FL designed, supervised the study and wrote the manuscript. All authors read and approved the final manuscript.

## Supplementary Material

Additional file 1 **Supplemental Figures TAT-α7pep2 peptide treatment has no effects on locomotor activity.** TAT-α7pep2 peptide treatment did not affect total distance travelled (Supplemental Figure1A), Margin Distance Travelled (Supplemental Figure1B), Margin Time Spent (Supplemental Figure 1C), Centre Distance Travelled (Supplemental Figure 1D), Centre Time Spent (Supplemental Figure 1E) in the open field test. Click here for file
