# Supplementary material for: α7nAchR/NMDAR coupling affects NMDAR function and object recognition
Source: Mol Brain. 2013 Dec 20;6:58. doi: 10.1186/1756-6606-6-58 (PMC3878138; doi:10.1186/1756-6606-6-58)
Supplement: Additional file 1 — Supplemental Figures TAT-α7pep2 peptide treatment has no effects on locomotor activity. TAT-α7pep2 peptide treatment did not affect total distance travelled (Supplemental Figure1A), Margin Distance Travelled (Supplemental Figure1B), Margin Time Spent (Supplemental Figure 1C), Centre Distance Travelled (Supplemental Figure 1D), Centre Time Spent (Supplemental Figure 1E) in the open field test. [file 1756-6606-6-58-S1.pptx]

## Slide 1
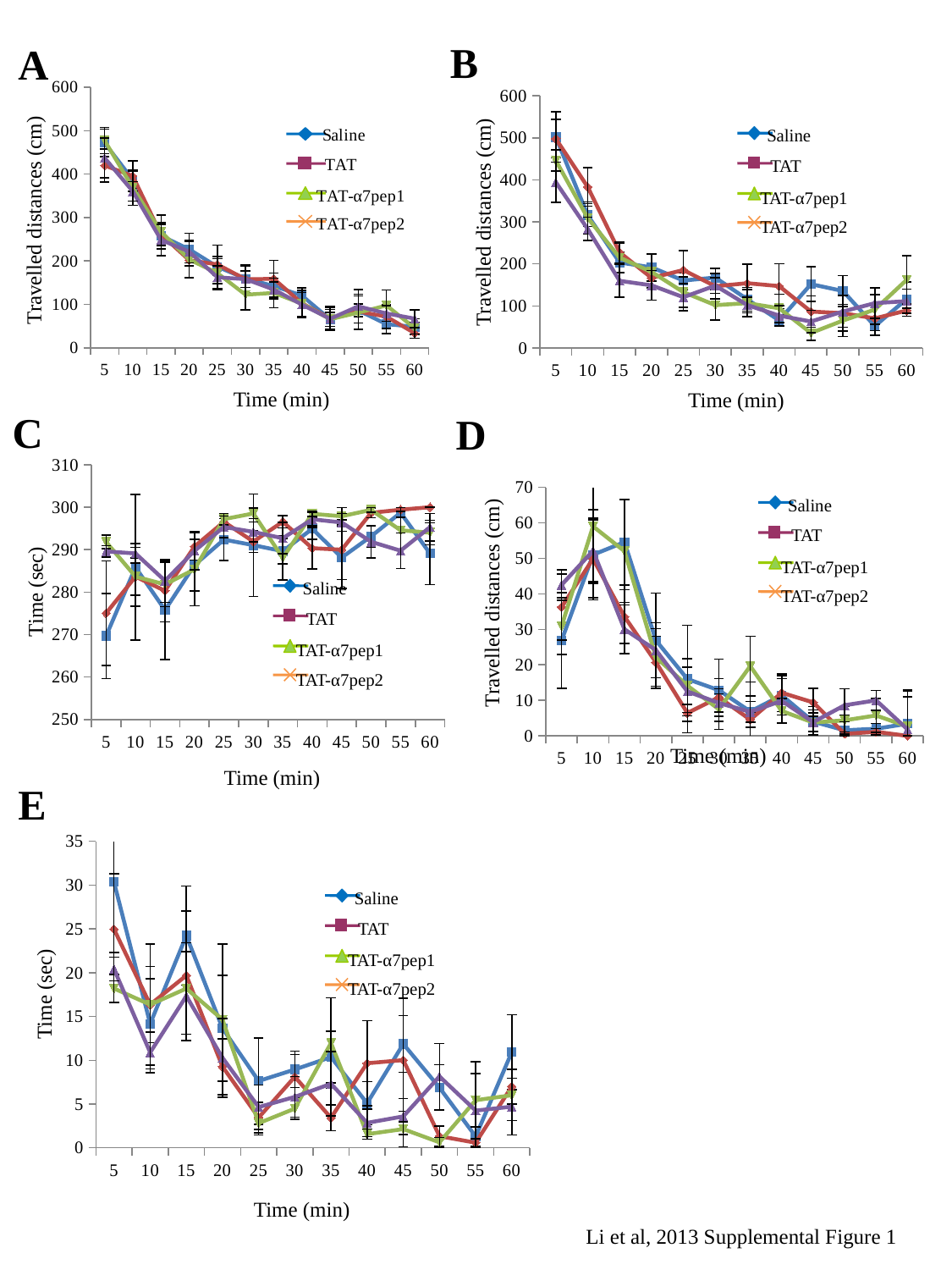

B
A
### Chart
| Category | Saline | TAT | TAT-α7pep1 | TAT-α7pep2 |
|---|---|---|---|---|
| 5 | 471.71428571428567 | 419.71428571428567 | 477.25 | 437.125 |
| 10 | 386.85714285714283 | 395.4285714285709 | 368.375 | 360.125 |
| 15 | 258.0 | 261.0 | 267.25 | 248.375 |
| 20 | 226.71428571428552 | 201.5714285714286 | 203.25 | 221.125 |
| 25 | 186.42857142857142 | 192.0 | 171.875 | 162.25 |
| 30 | 159.28571428571428 | 157.71428571428552 | 122.12499999999999 | 158.0 |
| 35 | 142.42857142857142 | 158.85714285714306 | 126.37499999999999 | 134.0 |
| 40 | 120.0 | 102.42857142857136 | 101.5 | 99.37499999999999 |
| 45 | 65.42857142857139 | 67.57142857142848 | 65.75 | 66.75 |
| 50 | 86.0 | 83.42857142857139 | 80.87499999999999 | 95.37499999999999 |
| 55 | 54.285714285714285 | 71.57142857142848 | 97.37499999999999 | 79.0 |
| 60 | 48.285714285714285 | 33.57142857142853 | 45.25 | 67.12499999999999 |Travelled distances (cm)
Time (min)
### Chart
| Category | Saline | TAT | TAT-α7-1 | TAT-α7-2 |
|---|---|---|---|---|
| 5 | 501.71428571428567 | 497.857142857143 | 446.25 | 394.5 |
| 10 | 316.0 | 383.285714285714 | 309.5 | 283.125 |
| 15 | 203.42857142857142 | 227.42857142857142 | 215.0 | 159.75 |
| 20 | 191.85714285714306 | 166.85714285714306 | 181.0 | 149.0 |
| 25 | 160.42857142857142 | 185.42857142857142 | 132.875 | 120.87499999999999 |
| 30 | 167.5714285714286 | 146.85714285714306 | 102.0 | 148.5 |
| 35 | 115.85714285714285 | 154.142857142857 | 106.75 | 102.37499999999999 |
| 40 | 65.71428571428572 | 147.28571428571428 | 94.62499999999999 | 77.0 |
| 45 | 151.85714285714306 | 86.71428571428572 | 35.75 | 63.125000000000036 |
| 50 | 135.5714285714286 | 82.85714285714282 | 65.25 | 86.87499999999999 |
| 55 | 52.285714285714285 | 70.42857142857139 | 91.5 | 106.75 |
| 60 | 116.42857142857136 | 89.285714285714 | 162.125 | 111.5 |Saline
TAT
TAT-α7pep1
TAT-α7pep2
Travelled distances (cm)
Time (min)
C
D
### Chart
| Category | Saline | TAT | TAT-α7-1 | TAT-α7-2 |
|---|---|---|---|---|
| 5 | 269.65714285714284 | 275.02857142857084 | 291.8 | 289.58750000000003 |
| 10 | 285.8428571428573 | 283.64285714285745 | 283.6500000000003 | 289.1125 |
| 15 | 275.77142857142894 | 280.31428571428575 | 281.81250000000006 | 282.675 |
| 20 | 286.35714285714283 | 290.72857142857094 | 285.36249999999995 | 289.7249999999997 |
| 25 | 292.37142857142857 | 296.5428571428573 | 297.1875 | 295.35 |
| 30 | 291.057142857143 | 291.8999999999997 | 298.52500000000003 | 294.1875 |
| 35 | 289.6428571428574 | 296.5714285714286 | 288.03749999999974 | 292.6875 |
| 40 | 294.91428571428565 | 290.3428571428574 | 298.4374999999996 | 297.15 |
| 45 | 288.1428571428573 | 289.985714285714 | 297.8625 | 296.41249999999974 |
| 50 | 293.12857142857104 | 298.6714285714285 | 299.375 | 291.85 |
| 55 | 298.7 | 299.41428571428565 | 294.5625 | 289.75 |
| 60 | 289.0857142857143 | 300.0 | 294.0 | 295.3 |Time (sec)
Time (min)
### Chart
| Category | Saline | TAT | TAT-α7-1 | TAT-α7-2 |
|---|---|---|---|---|
| 5 | 26.85714285714299 | 36.285714285714285 | 30.875 | 42.5 |
| 10 | 51.0 | 49.85714285714285 | 59.0 | 52.125000000000036 |
| 15 | 54.57142857142853 | 33.57142857142853 | 52.0 | 30.0 |
| 20 | 26.857142857142886 | 20.71428571428574 | 22.125 | 24.125 |
| 25 | 16.0 | 6.428571428571429 | 14.125 | 12.5 |
| 30 | 12.85714285714292 | 10.857142857142874 | 7.5 | 9.25 |
| 35 | 6.857142857142895 | 4.571428571428571 | 19.625 | 6.75 |
| 40 | 11.428571428571413 | 12.14285714285714 | 7.0 | 9.875000000000009 |
| 45 | 4.0 | 9.428571428571413 | 3.75 | 3.8749999999999987 |
| 50 | 1.5714285714286 | 0.5714285714285721 | 4.375 | 8.625 |
| 55 | 2.0 | 1.142857142857144 | 5.75 | 10.0 |
| 60 | 3.428571428571402 | 0.0 | 2.625 | 1.8125 |Travelled distances (cm)
Time (min)
Saline
TAT
TAT-α7pep1
TAT-α7pep2
Saline
TAT
TAT-α7pep1
TAT-α7pep2
E
### Chart
| Category | Saline | TAT | TAT-α7-1 | TAT-α7-2 |
|---|---|---|---|---|
| 5 | 30.342857142857145 | 24.97142857142857 | 18.2 | 20.412499999999977 |
| 10 | 14.157142857142915 | 16.35714285714284 | 16.349999999999987 | 10.887500000000006 |
| 15 | 24.22857142857139 | 19.68571428571427 | 18.1875 | 17.325 |
| 20 | 13.64285714285714 | 9.271428571428569 | 14.637500000000001 | 10.275 |
| 25 | 7.6285714285714255 | 3.457142857142858 | 2.812499999999997 | 4.6499999999999995 |
| 30 | 8.942857142857099 | 8.1 | 4.475 | 5.812499999999996 |
| 35 | 10.357142857142874 | 3.42857142857143 | 11.96250000000001 | 7.312499999999996 |
| 40 | 5.085714285714285 | 9.657142857142857 | 1.5625 | 2.8499999999999988 |
| 45 | 11.85714285714292 | 10.014285714285714 | 2.1375 | 3.5875 |
| 50 | 6.871428571428571 | 1.3285714285714287 | 0.6250000000000006 | 8.15 |
| 55 | 1.3000000000000003 | 0.5857142857142851 | 5.4375 | 4.25 |
| 60 | 10.914285714285716 | 7.0 | 6.0 | 4.7 |Time (sec)
Time (min)
Saline
TAT
TAT-α7pep1
TAT-α7pep2
Li et al, 2013 Supplemental Figure 1
